# Pre‐analytical stability of selected biochemical analytes in serum of horses and oxen stored at −20°C

**DOI:** 10.1002/vms3.1368

**Published:** 2024-02-15

**Authors:** Yoseph Cherinet Megerssa, Fikru Regassa Gari

**Affiliations:** ^1^ Department of Biomedical Sciences, College of Veterinary Medicine and Agriculture Addis Ababa University Bishoftu Ethiopia

**Keywords:** biochemical, horse, oxen, serum, storage

## Abstract

**Background:**

Delays between blood collection and analysis are inevitable, and samples are always stored in the refrigerator. The current study aimed to evaluate the stability of serum total cholesterol (TC), triglycerides (TG), total protein (TP), albumin and urea (URA) in horses and oxen after storage at −20°C.

**Methods:**

Sera from apparently healthy 20 male horses and 20 oxen were obtained and aliquots of serum were divided into 3 portions. The first tube was used for baseline (T0) measurement of analyte values, whereas the other two tubes, T1 and T2, were stored at −20°C for 1 and 2 months, respectively, and analyte measurement was done.

**Results:**

Results showed that the stability of TP (g/dL), URA (mg/dL) and TC (mg/dL) in oxen was statistically significant (*p* < 0.05). In horses, the stability of URA (mg/dL), TP (g/dL) and TG (mg/dL) were also statistically significant (*p* < 0.05). Additionally, URA and TC in oxen exceed TE_a_ following measurement at T2 and TG in horses following measurement at T1 and T2.

**Conclusion:**

Laboratories should consider the storage temperature and time for specific analytes among animals. Therefore, stability studies at various storage temperatures and times are recommended to fully validate the stability of the analytes.

## Graphical abstract

Blood clotted at room temp for 30 min, centrifugation at 1200 × *g* for 10 min to obtain serum. Serum was analysed at T0, T1 and T2 for TC, TP, ALB, TG and URA. TP, URA and TC in oxen and URA, TP and TG in horse showed *p* < 0.05. TEo for URA and TC in oxen > TE_a_ at T2, TEo for TG in horses > TE_a_ at T1 and T2.

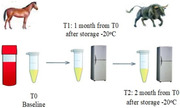

## 1 INTRODUCTION

Veterinary clinical laboratories play an important role in diagnostic and research services in medicine. Without the data from these laboratories animal disease detection, control and prevention would be significantly weakened (Edwards & Jeggo, 2012). Biochemical analysis is one of the important evaluations used to aid clinical diagnosis and research. Serum is the specimen of choice in many biochemical investigations due to no risk of anticoagulants interference (Taylor & Sethi, 2011). Numerous findings recognized that the pre‐analytical phase in biochemical testing is very sensitive to errors, most of which are made in this phase, primarily because of the difficulties in achieving standardized procedures including sample storage (Faulkner et al., 1990; Madira et al., 1993; Zhang et al., 1998).

In practice, it is common to re‐analyse the samples stored to confirm the previous results or to perform additional analysis; however, the stability of the analytes must be assured before notification of results, or before establishing new investigation (Felding et al., 1981). Clinicians also frequently ask to laboratory technologists to add tests on a previously collected specimen, making technologists with a dilemma: Is the analyte stable in the specimen during the storage time and temperature? In ambulatory clinical practice also there is an unavoidable delay between collection and testing, samples commonly being mailed to advanced laboratories (Moe et al., 2018).

Few studies described the effect of storage duration on the stability of biochemical constituents of serum in human including different animals (Banfi et al., 2002). However, information on the stability of commonly used biochemical analytes in horses and oxen serum stored at −20°C is limited and is absent in Ethiopia. Therefore, the current study examined the stability of five routine biochemical analytes, namely serum total cholesterol (TC), triglycerides (TG), total protein (TP), albumin(ALB) and urea (URA) following storage at −20°C at T1 (1 month) and T2 (2 months) with respect to baseline (T0).

## 2 MATERIALS AND METHODS

### 2.1 Study animals and sampling technique

The study animals were apparently healthy adult male horses, and oxen aged more than 4 years were recruited by convenient sampling technique at Society for Protection of Animal's Abroad (SPANA) Clinic and College of Veterinary Medicine and Agriculture Farm, respectively. Five millilitres of blood was collected from animals after having verbal informed consent from animal owners. Animals with a history of medication were ineligible due to the impact of treatment on biochemical analysis. The institutional animal research ethics committee at College of Veterinary Medicine and Addis Ababa University approved the ethical approaches of the study (Ref No VM/ERC/04/14/22).

### 2.2 Blood collection

Blood was collected from 20 horses and 20 oxen on separate days by a veterinarian from the jugular vein. The collected blood was left to clot at room temperature for 30 min. Serum was separated by centrifugation at 1200 × *g* for 10 min, and the sera were immediately transferred to respective test tubes. Aliquots of serum were then divided into three labelled Eppendorf Safe‐Lock tubes, and the two Eppendorf Safe‐Locks were stored at −20°C, whereas the other used immediately for day one (baseline) analysis. The samples were analysed for the TC (mg/dL), TP (g/dL), ALB (g/dL), TG (mg/dL) and URA (mg/dL).

### 2.3 Laboratory methods

The principles for the analysis of analytes include TC by CHOD‐PAP (cholesterol peroxidase 4‐aminophenazone), TP by biuret, ALB by bromocresol green, TG (GPO/PAP) and URA by kinetic urease/glutamate dehydrogenase (Burtis & Ashwood, 2001). The 20 samples were individually analysed for the different metabolites on day one (T0; baseline) immediately after centrifugation, whereas the other two groups T1 and T2 are stored at −20°C for 1 and 2 months, respectively. The samples stored at −20°C (T1 and T2) were analysed after bringing them to room temperature for approximately 1 h until completely thawed and then mixed properly with automatic pipettes before analysis. The chemistry analyser, EMP‐168 (AMP Diagnostics) and reagents from Jourilabs were used for the assay. Quality control (QC) samples of normal (N) and pathological (P) were run each day before analysis, and all QC values were within the expected QC range. The QC data CV for the serum TC, TG, TP, ALB and URA were 1.1, 2.3, 3.2, 1.5 and 1.8, respectively.

### 2.4 Data analysis and acceptable stability time

IBM SPSS version 20 statistical software was used to perform statistical analysis. Normality of the data distribution of the data was checked using the Kolmogorov–Smirnov test prior to statistical analysis. Data was analysed for mean and SD for T0, T1 and T2 values. A repeated measure ANOVA was used to compare the overall difference between the means at T0, T1 and T2. The results are expressed as means + SD. A *p*‐value of less than 0.05 was considered statistical significance. Post hoc analysis was assessed with a Bonferroni adjustment to ensure that a significance level at *p* < 0.017 (0.05/3 = 0.017) was set for each analyte regarding time intervals at T0, T1 and T2. For total allowable error (*T*_a_); bias (%) for T1 and T2 was calculated from percentage change from baseline (T0) values and CV (%) values from values obtained at T0, T1 and T2. TE_obs_ (%) = 2 × CV + bias (%). The TE_a_ (%) used in the current study was adopted from the American Society of Veterinary Clinical Pathology (ASVCP) guidelines for allowable total error guidelines for biochemistry. If TE_obs_ (%) is less than TE_a_ (%), the analytes are considered stable and no further action is needed (Harr et al., 2013).

## 3 RESULTS

In this study, repeated measures of ANOVA were used to describe the overall difference between the means at T0, T1 and T2. To this effect, Mauchly's test was done, and if *p* > 0.05, it meets the assumption of sphericity and its value taken. Although those violated the assumption of sphericity, *p*‐value from Greenhouse–Geisser correction was taken. As shown in the following, all parameters except TP and ALB in horses were not statistically significant (*p* > 0.05). ALB and TG in oxen were also not statistically significant (*p* > 0.05) (Table 1).

**TABLE 1:** Repeated measures of ANOVA values for analytes in horse and oxen.

			Change in biochemical analytes mean ± SD				
Analytes	Animal	*N*	T0	T1	T2	*F*‐value	*p*‐Value
Total protein (g/dL)	Horse	20	6.58 ± 0.57	6.56 ± 0.67	6.57 ± 0.74	0.038	0.899
	Oxen	20	5.73 ± 0.78	5.60 ± 0.73	5.63 ± 0.75	7.607	0.005^*^
Albumin (g/dL)	Horse	20	3.51 ± 0.29	3.50 ± 0.27	3.54 ± 0.25	0.487	0.568
	Oxen	20	3.26 ± 0.39	3.29 ± 0.43	3.28 ± 0.47	0.346	0.709
Urea (mg/dL)	Horse	20	49.26 ± 9.23	47.90 ± 9.58	47.46 ± 9.19	18.403	0.000*
	Oxen	20	47.78 ± 2.97	46.08 ± 3.76	45.15 ± 4.67	9.426	0.002*
Total cholesterol (mg/dL)	Horse	20	70.86 ± 11.73	70.28 ± 11.37	72.21 ± 12.13	4.934	0.027*
	Oxen	20	179.46 + 40.36	181.51 ± 41.15	185.36 ± 41.54	7.444	0.008*
Triglycerides (mg/dL)	Horse	20	33.81+18.90	37.79 ± 19.56	41.62 ± 20.61	70.367	0.000*
	Oxen	20	28.75+11.95	28.69 ± 8.74	29.74 ± 8.13	0.427	0.656

*indicates *p*‐Value < 0.05.

An attempt was made to further elaborate the effect of storage on the stability of analytes and a post hoc pairwise comparison by Bonferroni's correction showed that decreased stabilities of serum TP with statistical significance in oxen between T0 and T1 (5.73 vs. 5.60, respectively), *p* = 0.001 and between T0 and T2 (5.73 vs. 5.63, respectively), *p* = 0.005, were observed. In horses, decreased stabilities of URA with statistical significance between T0 and T1 (49.26 vs. 47.90, respectively), *p* = 0.001, and between T0 and T2 (49.26 vs. 47.46, respectively), *p* < 0.001, were noted. In oxen, decreased stabilities of serum URA with statistical significance between T0 and T1 (47.78 vs. 46.08, respectively), *p* = 0.001, and between T0 and T2 (47.78 vs. 45.15, respectively), *p* = 0.004 were noted.

In horses, variation in stability of serum TC was not statistically significant between T0 and T1 (70.86 vs. 70.28, respectively), *p* = 0.987, as well as between T0 and T2 (70.86 vs. 72.21 respectively), *p* = 0.347. In oxen, decreased stabilities of serum TC between T0 and T1 (179.46 vs. 181.51, respectively), *p* = 0.216, and statistical significance between T0 and T2 (179.46 vs. 185.36, respectively), *p* = 0.028, were noted. In horses, decreased stabilities of TG with statistical significance between T0 and T1 (33.81 vs. 37.79, respectively), *p* < 0.001, and between T0 and T2 (33.81 vs. 41.62 respectively), *p* < 0.001, were noted.

The study also examined the changes of these serums TC, TP, URA, TG and ALB after storage in terms of allowable error (TE_a_), which was adopted from the ASVCP guideline for allowable errors of biochemical analytes. Except for TC and URA in oxen and TG in horses, all analytes were TE_a_ (Table 2).

**TABLE 2:** Changes of analyte values after storage in terms of TE_a_ (%) in horse and oxen.

Analytes	Animal	*N*	Percentage change from baseline						
			T1			T2			
			CV	Bias	TE_o_	CV	Bias	TE_o_	TE_a_ (%)
Total protein (g/dL)	Horse	20	2.17	0.30	4.64	3.48	0.15	7.11	10
	Oxen	20	1.72	2.27	5.71	2.17	1.74	6.08	
Albumin (g/dL)	Horse	20	0.24	0.28	0.76	0.44	0.85	1.73	15
	Oxen	20	0.61	0.92	2.14	0.49	0.61	1.59	
Urea (mg/dL)	Horse	20	1.99	2.82	6.8	2.64	3.65	8.93	12
	Oxen	20	2.55	3.55	8.65	4.00	5.5	13.5	
Total cholesterol (mg/dL)	Horse	20	2.22	0.82	5.26	1.33	1.91	4.57	20
	Oxen	20	1.22	1.75	4.19	5.99	8.87	20.85	
Triglycerides (mg/dL)	Horse	20	7.87	11.77	27.51	14.65	3.3	32.6	25
	Oxen	20	0.15	0.21	0.51	2.39	5.5	10.28	

## 4 DISCUSSION

The general problem facing clinical laboratories is the integrity of specimens used for biochemical assays. A sample was considered to be significantly affected by the storage condition if the difference compared with the baseline values was outside allowable total error, and the difference was statistically significant (Tanner et al., 2008). The current study tries to address the stability of selected biochemical analytes following storage at −20°C for T1 and T2 with respect to T0. According to the findings of the current study, the stability of all analytes except TP in horses, TG in oxen and ALB in horses and oxen was statistically significant in terms of storage. In addition, URA and TC in oxen exceed the established TE_a_ for the analytes at T2. Triglycerides in horses also exceed the established TE_a_ for the analytes at T1.

The study is the first report in Ethiopia under veterinary laboratory settings that reported the stability of biochemical analytes in sera of horses and oxen following storage at −20°C. In literature, information is limited regarding the stability of biochemical analytes in animals. Reports which investigated the effect of storage on biochemical analytes in animals vary between species and are likely to be influenced by different laboratory methods (Jones, 1985). The term ‘instability’ of biochemical analytes does not always suggest decline in the level of the analytes, in some cases rather unexplained increases may also occur and the reasons for such changes need to be further elucidated (Quartey et al., 2018).

Similar to the current study, Cuhadar et al. (2013) noted variations of TP in sera when stored for 3 months. On the contrary, Kovačević et al. (2021) described the stability of TP for 6 months when stored at −20°C. A significant variation of URA in storage has been described by Cuhadar et al. (2013). On the other hand Kovačević et al. (2021) reported the stability of URA after 6 months of storage at −20°C. Paltiel et al. (2008) and Comstock et al. (2001) noted the stability of TC and TG contrary to this study. Kovačević et al. (2021) noted that TC and TG were stable at −20°C for 5 and 4 months, respectively. Similar to this study, the stability of ALB was noted by Akonde et al. (2008) over 5 weeks. Kovačević et al. (2021) also described the stability of ALB for 6 months when stored at −20°C. However, significant variability of ALB after storage at −20°C for 1–6 months was noted by Pawlik‐Sobecka et al. (2020).

Although the observed changes in the concentration of TP in oxen and URA and TC in horses are statistically significant, the potential clinical impact is not significant. This study has shown that most analytes were within an allowable limit except URA and TC in oxen at T2 and TG in horses at T1. In this regard, as no adequate literature in veterinary health care was found, the author is unable to discuss the findings of the current study in detail. The TE_a_ suggested described in ASVCP guidelines is general, and thus, it might not represent the allowable biological and analytical range for all species; hence, the determination of allowable total error across different animals might be needed.

## 5 LIMITATIONS OF THE STUDY

The study was unable to conduct stability studies in a wide range of temperatures and duration of time. Besides, the study includes some biochemical parameters due to the lack of test kits.

## 6 CONCLUSION

The current study showed that all analytes except TP in horse, TG in oxen and ALB in horse and oxen were stable for 2 months when stored at −20°C. However, further studies are necessary to assure any alterations in the results of biochemical analytes when stored at −20°C and at various storage temperatures and duration. In addition, the implementation of quality systems to achieve quality targets for TC and URA in oxen and TG in horses which shows greater TE_o_ as compared to the established TE_a_ is needed. In general, stability studies at different storage conditions and time intervals, across different animals, and laboratory methods are recommended.

## AUTHOR CONTRIBUTIONS

*Conceptualization; data curation; formal analysis; funding acquisition; investigation; methodology; project administration; resources; supervision; validation; visualization; writing – original draft; writing – review and editing*: Yoseph Cherinet Megerssa. *Conceptualization; data curation; formal analysis; funding acquisition; investigation; methodology; project administration; resources; validation; visualization; writing – original draft; writing – review and editing*: Fikru Regassa Gari.

## CONFLICT OF INTEREST STATEMENT

The authors declare that no conflicts of interest to declare.

## FUNDING INFORMATION

The Addis Ababa University research and technology transfer office through adaptive problem solving research fund (Reference no: RD/PY‐147/2021, Date 20‐September‐2021)

### PEER REVIEW

The peer review history for this article is available at https://www.webofscience.com/api/gateway/wos/peer-review/10.1002/vms3.1368.

## ETHICS STATEMENT

The study protocol obtained research ethical clearance approved by the institutional animal research ethics committee at the Addis Ababa University and College of Veterinary Medicine and Agriculture used in this study (Certificate reference no VM/ERC/04/14/022). Verbal informed consent was also obtained from the owners of animals to collect samples as suggested by institutional animal research ethics review committee.

## Data Availability

The data used to support this study are available from the corresponding author on request.
